# Cardiovascular involvement in Erdheim–Chester diseases is associated with myocardial fibrosis and atrial dysfunction

**DOI:** 10.1007/s11547-023-01616-7

**Published:** 2023-03-22

**Authors:** Anna Palmisano, Corrado Campochiaro, Davide Vignale, Alessandro Tomelleri, Giacomo De Luca, Elisa Bruno, Caterina B. Monti, Giulio Cavalli, Lorenzo Dagna, Antonio Esposito

**Affiliations:** 1 Clinical and Experimental Radiology Unit, Experimental Imaging Center, IRCCS San Raffaele Scientific Institute, Via Olgettina 58 – 60, 20132 Milan, Italy; 2 School of Medicine, Vita-Salute San Raffaele University, Milan, Italy; 3 Unit of Immunology, Rheumatology, Allergy and Rare Diseases, IRCCS San Raffaele Scientific Institute, Milan, Italy; 4 Department of Biomedical Sciences for Health, Università Degli Studi Di Milano, Milan, Italy

**Keywords:** Magnetic resonance imaging, Erdheim–Chester disease, Fibrosis, Myocardial infarction, Edema, Cardiac

## Abstract

**Purpose:**

Erdheim–Chester disease (ECD) is a rare multisystem histiocytosis, whose cardiovascular involvement has not been systematically characterized so far. We aimed to systematically (qualitatively and quantitatively) describe the features of cardiovascular involvement in a large cohort of ECD patients and to evaluate its impact on myocardial fibrosis extension and cardiac function.

**Material and methods:**

Among 54 patients with biopsy-proven ECD, 29 patients (59 ± 12 years, 79% males) underwent 1.5-T CMR using a standardized protocol for qualitative and quantitative assessment of disease localization, evaluation of atrial and ventricular function, and assessment of non-dense and dense myocardial fibrosis.

**Results:**

The right atrioventricular (AV) groove was the most commonly affected cardiac site (76%) followed by the right atrial walls (63%), thoracic aorta (59%), and superior vena cava (38%). Right AV groove involvement, encasing the right ventricular artery, was associated with non-dense myocardial fibrosis in the infero-septal (20/26 patients) and the inferior (14/26 patients) mid-basal left ventricular (LV) wall. In two patients with right AV groove localization, LGE revealed myocardial infarction in the same myocardial segments. Three out of five patients with left AV groove involvement had non-dense LGE on the lateral LV mid-basal wall. Bulky right atrial pseudomass was associated with atrial dysfunction and superior and inferior vena cava stenosis.

**Conclusions:**

In ECD patients, AV groove localization is associated with LV wall fibrosis in the downstream coronary territories, suggesting hemodynamic alterations due to coronary encasement. Conversely, atrial pseudomass ECD localizations impact on atrial contractility causing atrial dysfunction and are associated with atrio-caval junction stenosis.

## Introduction

Erdheim–Chester disease (ECD) is a rare systemic non-Langerhans cells histiocytosis of unknown etiology characterized by multiorgan xanthomatous infiltration by CD68 + , CD1a − foamy histiocytes (1,2). Its clinical presentation is heterogeneous, ranging from isolated bone involvement to multisystemic and sometimes life-threatening phenotypes (1,2). Bone pain due to skeletal infiltration is the most common clinical manifestation, occurring in approximately 95% of patients. Involvement of central nervous system (CNS) and of cardiovascular system are independent predictors of fatal outcome [1, 3, 4]. Specifically, in patients with cardiac localization, mortality rate at 3 years is 60% [1, 4].

Cardiovascular involvement in ECD is poorly characterized for scarcity of data from systematic screenings with advanced imaging modalities. Available data are mainly derived from case reports [5, 6] or small case series with heterogeneous imaging evaluation [4, 7]. Cardiac involvement is estimated to occur in 50–70% [8, 9] of patients. The most common findings are pericardial infiltration with effusion [2] and right atrioventricular groove pseudo-tumoral masses [4, 7, 10, 11]. Although cardiac ECD can be asymptomatic, some patients develop arrhythmias, valvular heart disease, ischemia, or heart failure [12]. Therefore, given its prognostic impact, a deeper understanding of the cardiac functional and structural impairment in ECD is of fundamental importance for risk stratification and therapeutic management.

Cardiac magnetic resonance (CMR) is the imaging modality of choice for morphofunctional assessment of the cardiac chambers and for the identification of ECD involvement for its superior soft tissue resolution [13–15].

The aim of the present study is to qualitatively and quantitatively characterize ECD cardiac involvement, and to investigate the impact of site and extension of ECD cardiac involvement on cardiac chamber volumes, function, and structural remodeling.

## Materials and methods

### Study population

This observational retrospective study includes 54 consecutive patients with biopsy-proven ECD referred to our Institution (IRCCS San Raffaele Hospital, Milan, Italy) between June 2009 and December 2021.

In previous studies were included data on 25 [16] and 1 of 56 patients [17]. These previous articles dealt with treatment efficacy.

**Fig. 1:**
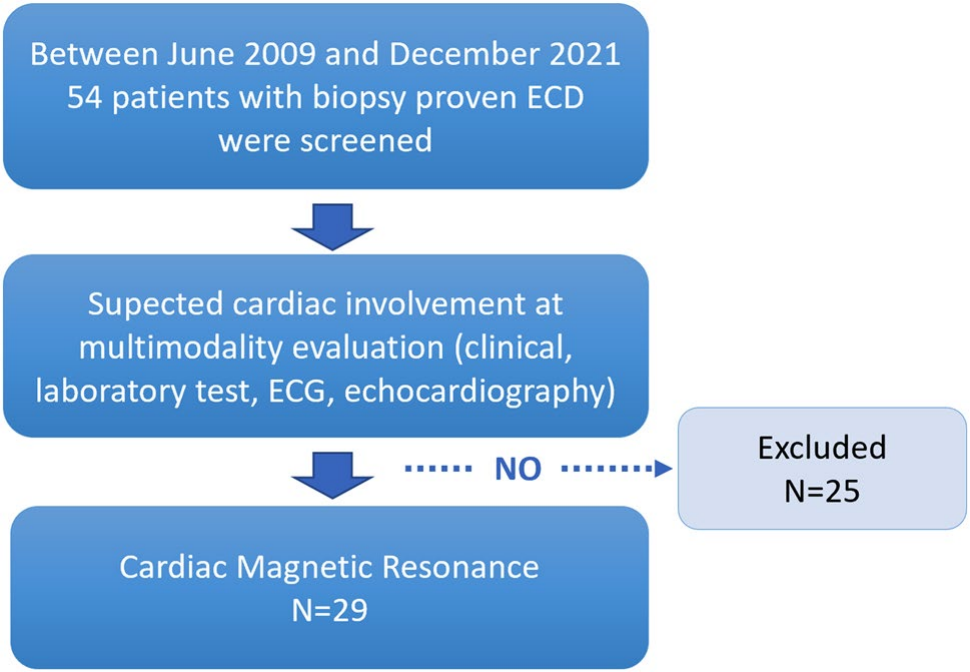
Enrollment flowchart

In the present study we included all patients with clinical suspicion of cardiac involvement at multimodality evaluation (clinical evaluation, laboratory test, electrocardiogram, and echocardiography) who underwent CMR for disease confirmation and characterization before therapy start (Fig. 1). Only 29 of 54 patients met this inclusion criteria and were included in the present study. The study was approved by the Ethic Committee and all patients signed informed consent.

### CMR protocol

CMR was performed with 1.5-T system (Achieva dStream, Philips Medical Systems, Eindhoven, the Netherlands) using a 32-element cardiac phased-array coil. T2-weighted short-tau inversion recovery (T2-STIR) sequences were acquired using a body coil. All images were acquired during breath-holding periods. CMR protocol included: multiplanar (axial, sagittal and coronal planes) 2-D balanced turbo field echo (BTFE) images and axial and sagittal T2-STIR and proton density (PD) sequences covering the entire mediastinum; steady-state free precession (SSFP) cine, T2-STIR, black-blood PD, PD-fat-saturated (PD-fat-sat) sequences and 10-min post-contrast (0.15 mmol/kg of gadobutrol: Gadovist, Bayer Healthcare, Berlin, Germany) three dimension inversion recovery T1w (3D-IR-T1w) images acquired in the short-axis, 2-chamber and 4-chamber long-axis cardiac views.

In a subgroup of ten patients who underwent CMR after 2016 T1 mapping acquisition was performed. Both native and post-contrast T1 mapping were acquired in the same three short-axis planes (base, mid-ventricle, apex) and in a single four chambers long-axis view, using a modified Look-Locker inversion recovery sequence with 5(3)3 and 4(1)3(1)2 sampling scheme, respectively.

### CMR analysis

All CMRs were analyzed by a cardiac radiologist with 10 years of experience in cardiac imaging and revised by a senior cardiac radiologist with 17 years of experience using a dedicated cardiac analysis software (CVI42v.5.13.5, Circle Cardiovascular Imaging, Calgary, AB, Canada).

Left and right ventricle end-diastolic and end-systolic volumes (EDV, ESV), ejection fractions (EF) and myocardial mass were extracted from automatic segmentation of endocardial and epicardial contours in the end-diastolic and end-systolic short-axis cine-SSFP images, and manually corrected when necessary.

Atrial ESV, EDV and EF were assessed by biplane area-length method after semiautomatic tracing of the endocardial contours in 2-chambers and 4-chambers long-axis views.

Myocardial edema was assessed on T2-STIR images as areas characterized by relative hyperintensity at visual assessment and by the measurement of T2-ratio (positive ≥ 1.9). The SI of myocardium resulted from semiautomatic contouring of endocardial and epicardial left ventricular borders, while the SI of the reference skeletal muscle resulted from a manual ROI of at least 10 mm^2^ placed in a chest wall muscle.

Myocardial fibrosis was assessed as Late Gadolinium enhancement (LGE) on 3D-IR-T1w images and classified as non-dense scar (signal intensity ≥  + 3SD on normal myocardium) and dense scar (signal intensity ≥ 5SD on normal myocardium). Both non-dense and dense scar were quantified as percentage of LV wall mass. Additionally, LGE was classified according to the transmural pattern (sub-endocardial, mid-wall, sub-epicardial or transmural) and the segmental (AHA 16 segments model) involvement.

Native T1 mapping and ECV values were obtained after outlining endocardial and epicardial edges on the short-axis slices (base, mid-ventricle, apex), with an automatic offset of 10% from endocardial and epicardial borders to reduce partial volume effect. Local reference value were for native T1 ≤ 1045 ms, and for ECV < 27%.

Site of cardiovascular involvement was firstly visually assessed on axial PD, BTFE and LGE images, and then quantified on the sequence showing the best image quality, by tracing contours slice-per-slice in order to cover the entire mass and compute the mass volume. Additionally, maximum mass thickness was manually measured on axial images.

Pericardial effusion and pericardial thickening were visually identified and manually measured at the thickest point. Similarly, aortic wall infiltration was visually identified and manually measured at the thickest point.

Vascular stenosis or dilatation at the level of ECD infiltration were assessed as vascular diameter change in comparison to a reference vascular diameter measured at a level without ECD infiltration.

### Statistical analysis

Data analyses were performed using IBM SPSS for macOS version 28.0.1.0 (IBM Corp., Armonk, NY). Data are reported as medians and interquartile ranges [IQRs] or frequencies and relative percentages. Mann–Whitney U test for unpaired samples was used to compare continuous variables. A value of *p* ≤ 0.05 was considered statistically significant.

## Results

### Study population

Patients were mainly men (M:F = 23:6), with a median age of 61 [IQR, 52–65] years. All patients had skeletal involvement (Table 1). About two-third of patients had at least one of the following central nervous system signs (20 of 29; 69%): diabetes insipidus (18 of 20, 90%), cerebellar ataxia (9 of 20, 45%) or papilledema (3 of 20, 15%). Exophthalmos, diplopia, or visual impairment were present in 14 subjects (48%), as signs of retro-orbital involvement. Retroperitoneal space infiltration, and consequent renal involvement, was found in 15 patients (53%), mainly manifesting as chronic kidney failure (13 of 15, 86%). In one-half of patients (15 of 29; 52%) there was radiological evidence of pulmonary involvement, mainly with few symptoms such as cough or chest discomfort (Table 1).

**Table 1:** Patient Extra-cardiac Characteristics

Patient clinical feature	Value
Male participants (n)	23/29 (79%)
Median age at CMR examination (y)	61 [52–65]
Median age at diagnosis (y)	57 [45–59]
Diagnostic delay (y) *	5 [4.5–6.5]
*Organ involvement*	
Skeleton (n)	29/29 (100%)
CNS (n)	20/29 (69%)
Kidneys (n)	15/29 (52%)
Lungs (n)	15/29 (52%)
Eyes (n)	14/29 (48%)
Skin (n)	11/29 (38%)

Data are expressed as mean ± standard deviation (SD) or frequencies when indicated

*Time interval between onset of symptoms and biopsy-confirmed diagnosis of Erdheim–Chester disease

### CMR findings: cardiovascular localization site and extent

**Fig. 2:**
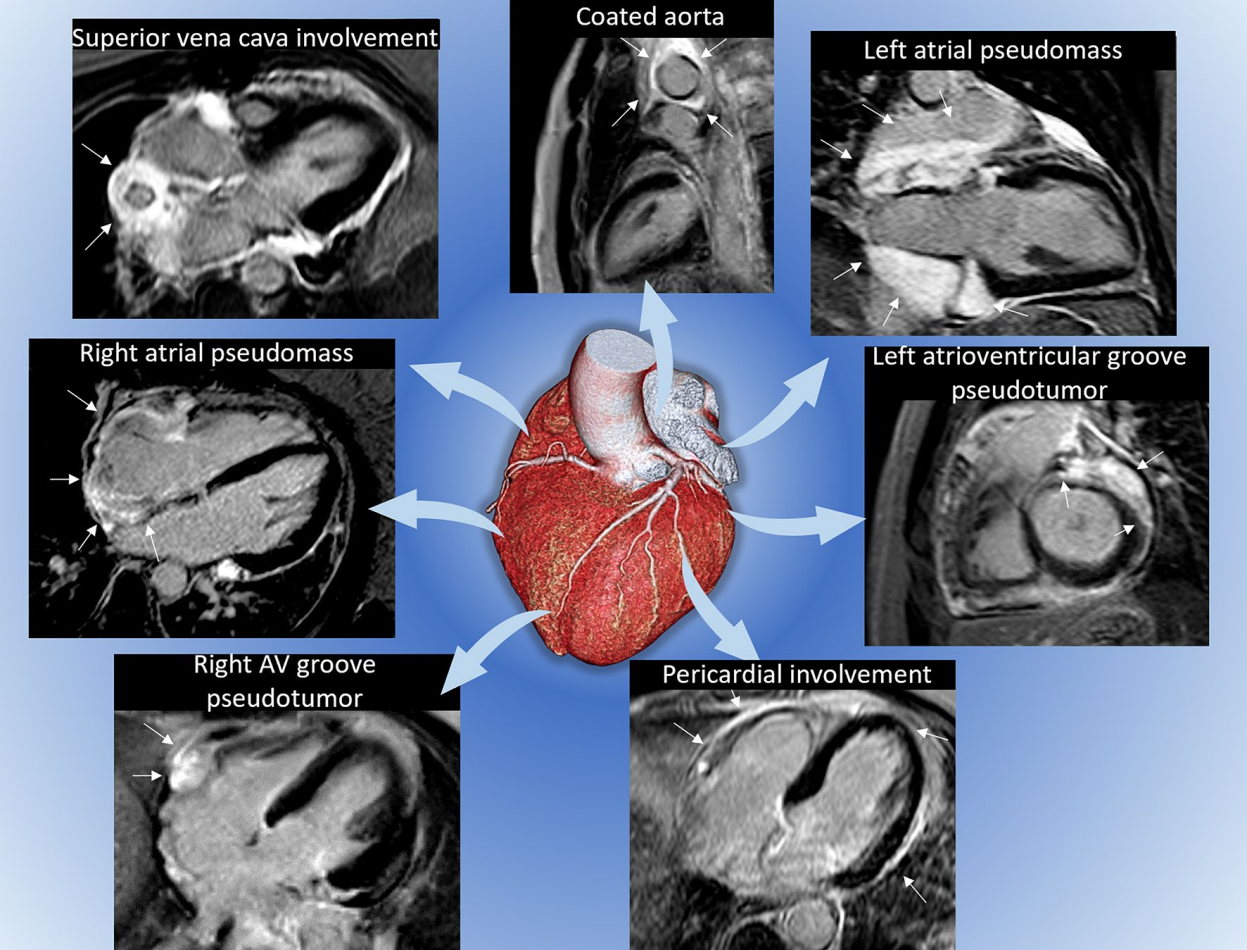
The spectrum of cardiovascular localization of Erdheim–Chester disease found at CMR. LGE images of most typical ECD cardiac localization. In each image as in each patient more findings may coexist; however, the main finding is identified by arrows

Three out of 29 patients had chronic kidney failure and CMR was performed without contrast media. Cardiovascular involvement was found in 22 of 29 patients (76%). Detailed information about cardiovascular localization of ECD at CMR is reported in Table 2 and Fig. 2.

**Table 2:** CMR findings: cardiovascular localization site and amount

Site and amount of cardiac ECD involvement	Number of patients (n)	Value
Overall cardiac ECD involvement (mL)	22/29 (76%)	32 [14–99]
Right atrioventricular groove	22/22 (100%)	19 [12–46]
RA walls (RA pseudomass)	14/22 (63%)	21 [6–38]
Left atrioventricular groove	5/22 (23%)	6 [4–10]
Anterior interventricular groove	4/22 (18%)	8 [7.5–9.5]
LA walls (LA pseudomass)	1/22 (4%)	8.7
Pericardial thickening (mm)	16/29 (55%)	22.5 [8.8–33.5]
Pericardial effusion (mL)	14/29 (48%)	88 [44–171]
Aortic wall infiltration (mm)	17/29 (59%)	8 [6–9]
Maximum aortic root caliper (mm)	29/29	33 [30–35]
Maximum ascending aorta caliper (mm)	29/29	32 [30–35]
Maximum aortic arch caliper (mm)	29/29	27 [24–29]
Maximum descending aorta caliper (mm)	29/29	23 [21–24]
Coated aorta	9/29 (31%)	
Superior vena cava infiltration (mL)	11/29 (38%)	8 [6–22]
Minimum vessel caliper (mm)		12 [9–14]
Vessel stenosis (%)		45 [31–51]
Inferior vena cava infiltration (mL)	7/29 (24%)	13 [7–15]
Minimum vessel caliper (mm)		21 [16–25]
Vessel stenosis (%)		12 [5–28]

Data are expressed as medians and IQRs or frequencies when indicated

*ECD* Erdheim–Chester disease, *LA* Left Atrium, *RA* Right Atrium, *AV* Atrioventricular

ECD cardiovascular localization at CMR was characterized by intermediate signal in PD images, mild hyperintensity in PD-fat-sat, and high signal intensity in T2-STIR and LGE images and were present in 22/29 patients (76%).

Right AV groove localization (median mass volume: 19 [IQR, 12–46] mL, maximum thickness: 16 [IQR, 16–27] mm) was present in all patients with cardiovascular involvement. Right atrial wall infiltration was found in 14 of 29 patients (48%; median mass volume: 21 [IQR, 6–38] mL, maximum thickness: 16 [IQR, 15–20] mm), and had always a pseudotumor appearance. Five patients (17%) had also left AV groove infiltration (median amount of mass: 6 [IQR, 4–10] mL).

Pericardial late enhancement was present in 16 patients (55%; pericardium median thickness: 2.5 [IQR 2–3.3] mm) and was associated with pericardial effusion in 14 of them (median thickness: 10 mm [IQR 7–20] mm). In eight patients it had a circumferential distribution. Thoracic aorta was the most affected mediastinal vessel (17 of 29, 59%), with maximum wall thickness of 20 mm (median value: 8 [IQR, 6–9] mm). In nine patients (31%), its involvement had the appearance of “coated aorta” [18]. No patient had lumen stenosis. Superior and inferior atrio-caval junctions were affected in 38% (11/29) and 24% (7/29) of patients, respectively. Median severity of stenosis was 45% [IQR, 31–51] for superior atrio-caval junction involvement and 12% [IQR, 5–28] for inferior atrio-caval junction involvement.

The overall volume of ECD cardiovascular localizations was 32 [IQR, 14–99] mL and it correlated with the number of anatomical structures involved (rho: 0.886, *p* < 0.001).

### CMR findings: myocardial characteristics and remodeling

**Fig. 3:**
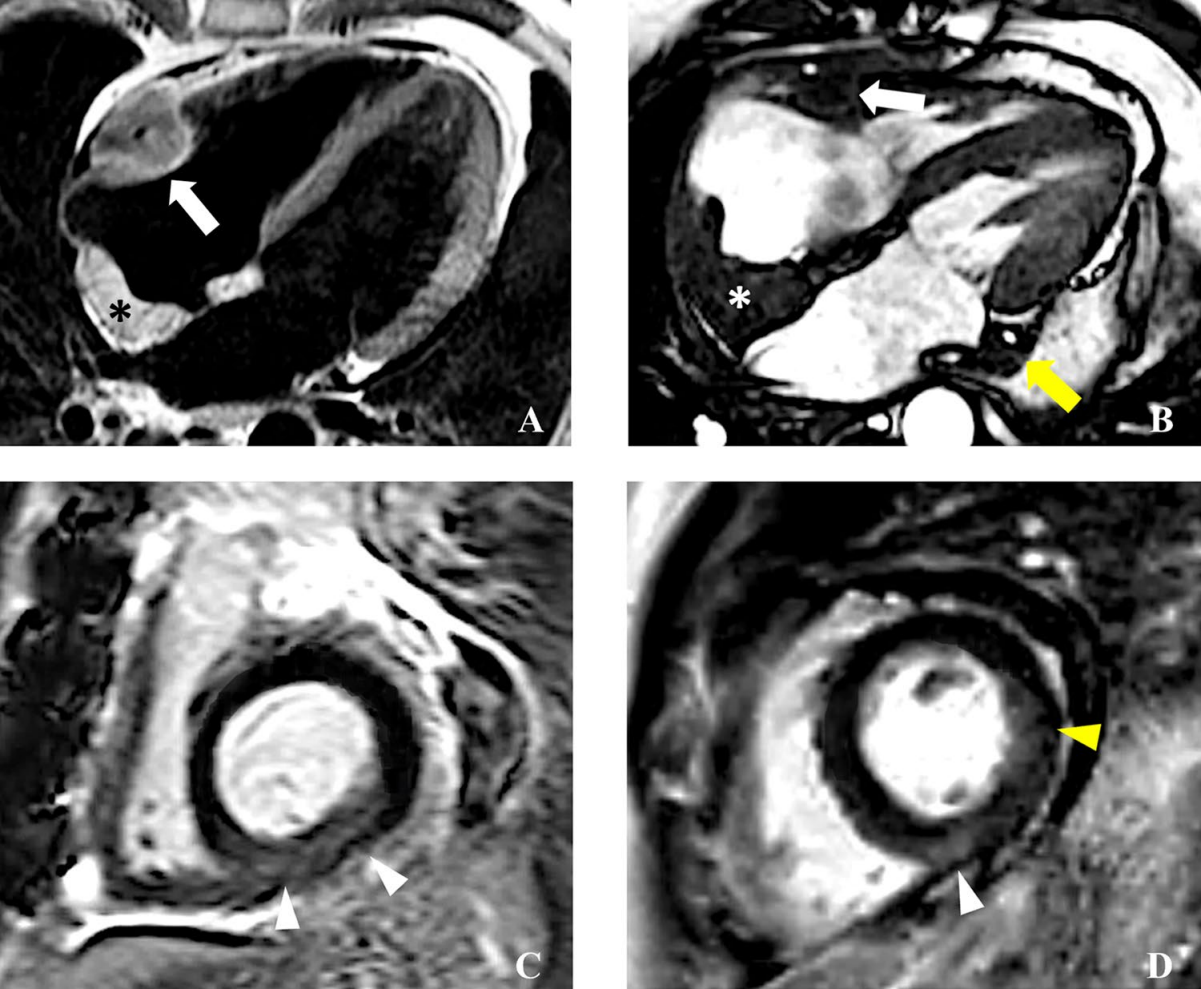
CMR findings of atrioventricular groove infiltration with coronary encasement and non-dense LGE with coronary distribution. In **A**, **B** are reported two cases of patients with cardiac ECD localization characterized by bulky right atrioventricular pseudotumors of 21 ml (arrow in A) and 46 ml (arrow in **B**), respectively. Corresponding LGE images in C-D showed myocardial enhancement involving the basal infero-septal and inferior segments (arrows in **C**) in the first case and inferior and lateral basal segments in D. In both the cases the LGE had transmural non-dense aspect, accounting for 7% and 6% of myocardial mass at quantitative (+ 3SD) LGE analysis, respectively. In cases **A** and **B** right atrial involvement coexist (asterisk). Notably in **B** was also present an involvement of the left atrioventricular groove with left circumflex artery encasement (yellow arrow in **B**) which resulted associated to a non-dense myocardial fibrosis also in the lateral basal segment (yellow arrows in **D**)

**Fig. 4:**
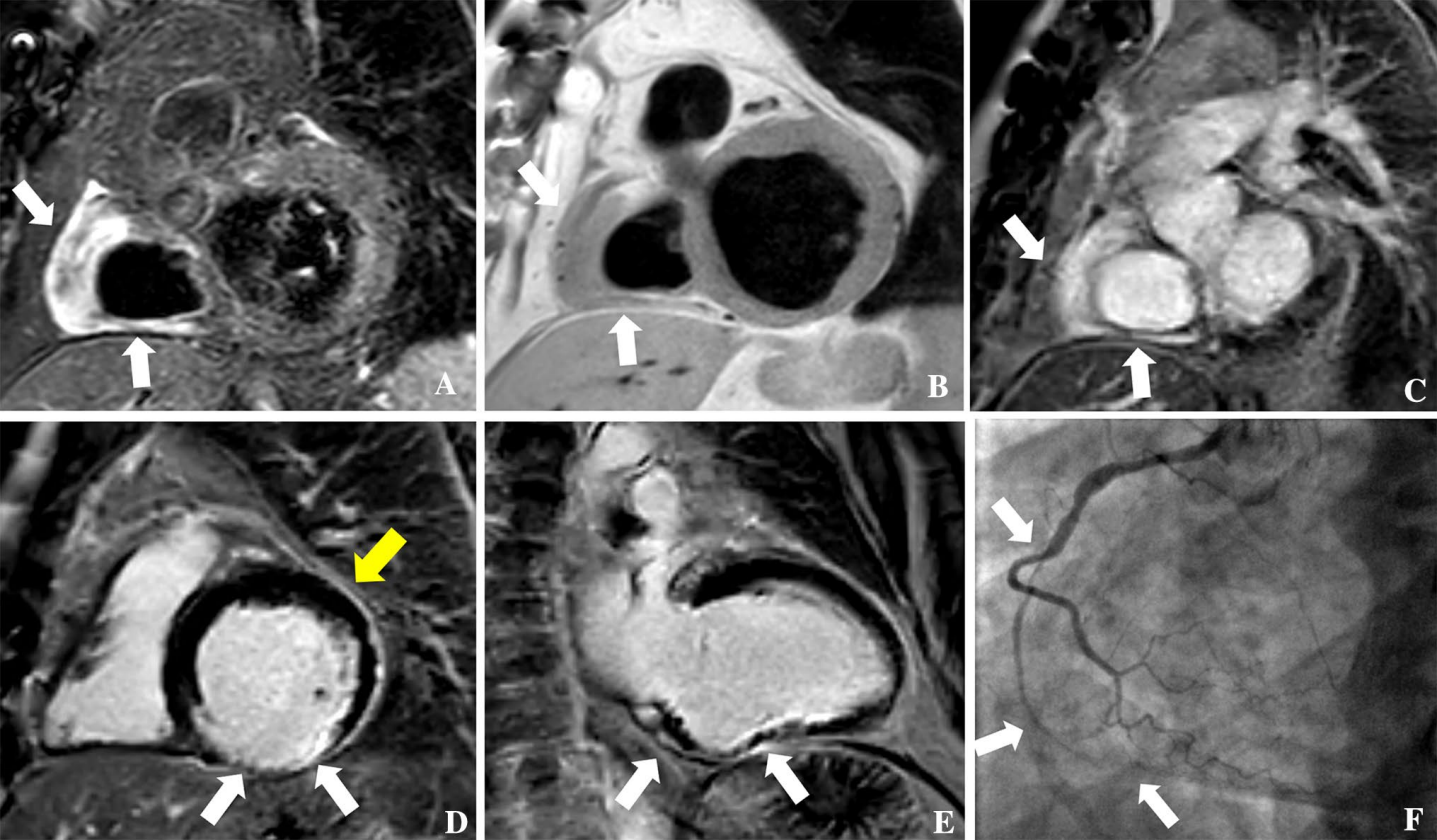
CMR in a patient with right atrioventricular groove infiltration and right coronary stenosis and inferior left ventricle infarction. A 54 years old male underwent CMR showing right atrioventricular groove pseudomass (33 cc) with right coronary encasement with typical signal intensity features suggestive for ECD localization, as brightness in STIR (arrow in **A**), and LGE (arrow in **C**) images and with intermediate signal intensity in PD (arrow in **B**) that was associated the transmural infarction involving the inferior left ventricle wall (white arrows in **D** and **E**). Moreover, diffuse pericardial thickening suggestive for pericardial involvement in absence of significant pericardial effusion was present (yellow arrow in **D**). The patient underwent invasive coronary angiography which showed diffuse severe reduction of right coronary artery caliper in site of right atrioventricular groove localization

Main myocardial findings at CMR are reported in Table 3. Ventricles’ volumes and ejection fraction were globally in the range of normality (LVEDV/BSA: 63 [IQR, 53.5–74.7] mL/m^2^; RVEDV/BSA: 60 [IQR, 52–66] mL/m^2^; LVEF: 64 [IQR, 60–66] %; RVEF: 62 [IQR, 55–69] %), as well as the end-diastolic LV wall mass (LV-EDWM/BSA: 52 [IQR, 46–64] mL/m^2^). Myocardial T2-ratio was slightly increased in patients with ECD cardiac involvement compared to those without cardiac involvement (2.1 [IQR, 2–2.2] vs 1.65 [IQR, 1.6–1.7]), *p* < 0.001). This finding indicates a slight diffuse myocardial edema in the former group. All participants except one (25 of 26; 95%) had non-dense LGE (between + 3 and + 5 SD), with a median non-dense scar burden of 5.8 (IQR, 4.2–8.1)%. Non-dense fibrosis had a transmural pattern in most cases (22 of 26; 84%), mostly involving the infero-septal mid-basal wall (20 of 26; 77%), the inferior wall (14 of 26; 54%), and the lateral mid-basal wall (3 of 26; 11%) (Fig. 3). LGE in the infero-septal and inferior mid-basal wall was always associated with right AV groove involvement and right coronary artery encasement (Fig. 3A–C). Lateral LGE was associated with left AV groove ECD involvement (Fig. 3B,D). Specifically, three out of five patients (60%) with left AV groove involvement had lateral LGE, while no patient without left AV groove involvement had lateral LGE. Among patients with evidence of LGE, only two had large dense scars (≥ + 5SD) with transmural involvement, suggestive for inferior myocardial infarction (Fig. 4) involving 6% and 12% of LV myocardial wall, respectively, associated with right AV pseudomasses of 23 mL and 34 mL, respectively. One of these two patients underwent invasive coronary angiography showing a diffuse reduction of right coronary artery caliber (Fig. 4).

**Table 3:** CMR findings: myocardial characteristics and remodeling

Functional Parameters	Number of patients (n)	Value	*p* value
LV EDV (mL), LV EDV/BSA (mL/m^2^)	29/29	120 [95–149], 63 [53.5–74.7]	–
LV EF (%)	29/29	64 [60–66]	–
RV EDV (mL), RV EDV/BSA (mL/m^2^)	29/29	112 [90–133], 60 [52–66]	–
RV EF (%)	29/29	62 [55–69]	–
LV EDWM (g), LV EDWM/BSA (g/m^2^)	29/29	97 [83–122], 52 [46–64]	–
LA ESV (mL), LA ESV/BSA (mL/m^2^)	29/29	55 [43–81], 28 [24–42]	–
LA EDV (mL), LA EDV/BSA (mL/m^2^)	29/29	29 [18–37], 12 [10–17]	–
LA EF (%)	29/29	54 [46–63]	–
Overall RA ESV (mL)	29/29	58 [41–68)	
Patients with RA pseudomass	14/29 (48%)	57 [35–71]	n.s
Patient without RA pseudomass	15/29 (52%)	56 [43–66]	
Overall RA ESV/BSA (mL/m^2^)	29/29	27.7 [21–36]	
Patients with RA pseudomass	14/29 (48%)	28 [21–36]	n.s
Patient without RA pseudomass	15/29 (52%)	25 [22–36]	
Overall RA EDV (mL)	29/29	22 [15–40]	
Patients with RA pseudomass	14/29 (48%)	40 [12–49]	0.02
Patient without RA pseudomass	15/29 (52%)	20 [17–25]	
Overall RA EDV/BSA (mL/m^2^)	29/29	12 [10–17]	
Patients with RA pseudomass	14/29 (48%)	10.6 [7–12]	0.04
Patient without RA pseudomass	15/29 (52%)	18 [10–25]	
Overall RA EF (%)	29/29	53 [41–67]	
Patients with RA pseudomass	14/29 (48%)	45 [29.7–60]	0.03
Patient without RA pseudomass	15/29 (52%)	56 [52–69]	
*T2 STIR images: T2 ratio*			
Patient with cardiac ECD involvement	22/29	2.1 [2–2.2]	< 0.001
Patient without cardiac ECD involvement	7/29	1.65 [1.6–1.7]	
*Quantitative LGE assessment (% of injured myocardial mass)*			
Overall 3SD LGE	26/29	5.8 [4.2–8.1]	
Patient with AV groove involvement	21/26 (80%)	6.7 [5.1–9]	0.003
Patients without AV groove involvement	5/26 (19%)	1.2 [1, 2]	
Overall 5SD LGE	26/29	1.1 [0.5–1.8]	
Patient with AV groove involvement	21/26 (80%)	1.2 [0.7–1.9]	n.s
Patients without AV groove involvement	5/26 (19%)	0.2 [0.2–0.4]	
*Qualitative LGE assessment*			
LGE pattern			
Transmural	22/26 (84%)		
Mid-wall	3/26 (11%)		
Sub-epicardial	0/26		
Sub-endocardial	0/26		
LGE distribution			
Infero-septal wall AHA segments: 3; 9	20/26 (77%)		
Inferior wall AHA segments: 4; 10; 15	14/26 (54%)		
Infero-lateral wall AHA segments: 5; 11	3/26 (11%)		
*T1 mapping*			
Native myocardial T1 (ms)	10/29 (34%)	1059 [1044–1085]	
Patient with ECD localization	7/10	1067 [1059–1098]	0.04
Patient without ECD localization	3/10	1027 [1019–1042]	
ECV (%)	10/29 (34%)	30 [28–31]	
Patient with ECD localization	7/10	31 [30–32]	0.017
Patient without ECD localization	3/10	26 [26, 27]	

Data are expressed as medians and IQRs or frequencies when indicated

*ECD* Erdheim–Chester disease, *RV* Right ventricle, *LV* Left Ventricle, *EDV* End-Diastolic Volume, *ESV* End-Systolic Volume, *BSA* Body Surface Area, *EF* Ejection Fraction, *EDWM* End-Diastolic Wall Mass, *LA* Left Atrium, *RA* Right Atrium, *LGE* Late Gadolinium Enhancement, *AHA* American Heart Association, *ECV* Extracellular Volume, *AV* Atrioventricular, *SD* Standard Deviation

**Fig. 5:**
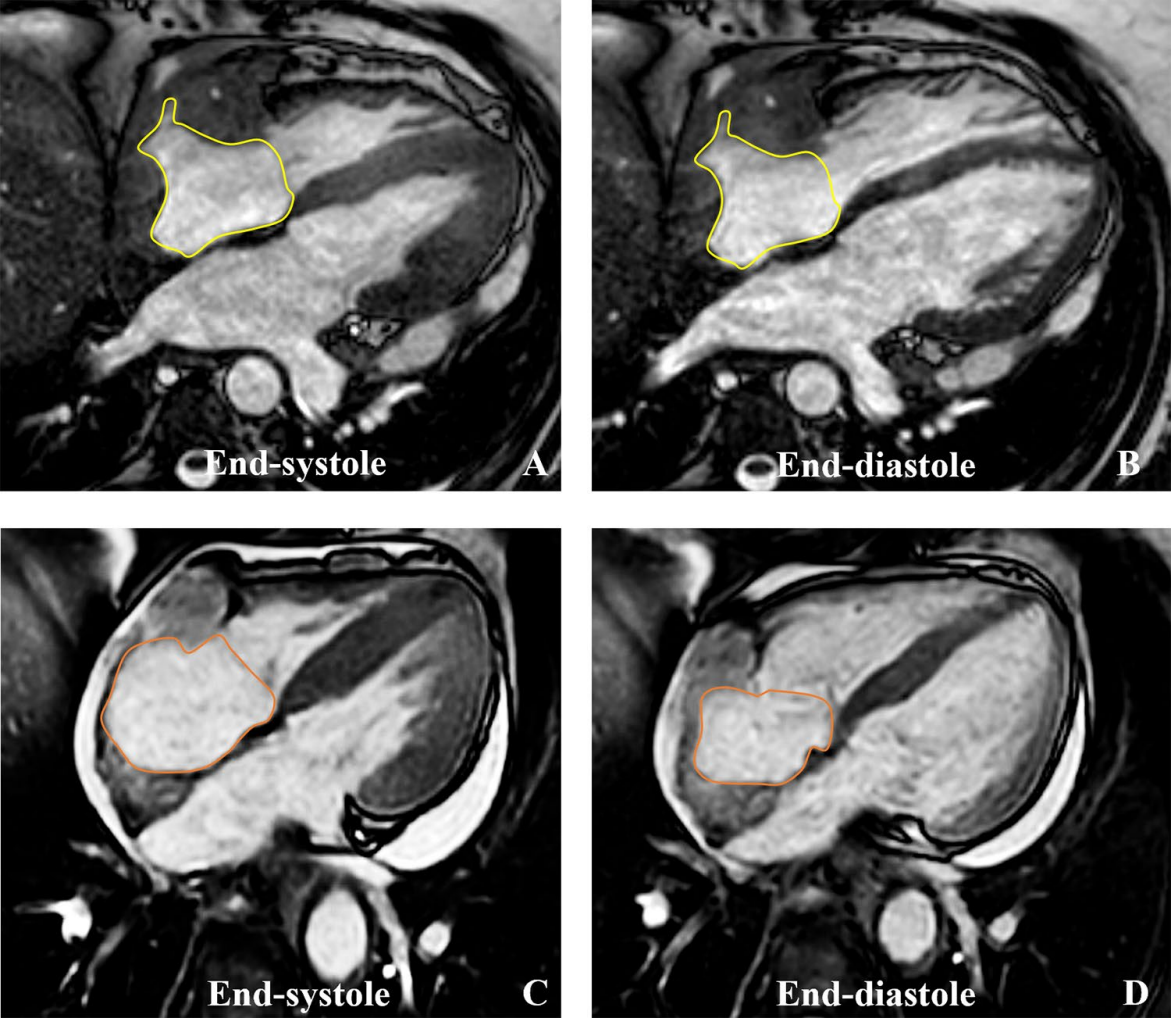
CMR images in two patients with right atrial pseudomasses with (**A**, **B**) and without (**C**, **D**) atrial dysfunction. In A-B are reported end-systolic and end-diastolic 4CH view cine-SSFP images of a 64 years old male patient presenting with huge ECD cardiac involvement (total localization volume 137 ml) and huge right atrial pseudomass (volume 42 ml) with extensive infiltration of posterior and free wall of the right atrium causing atrial wall stiffness and limited systo-diastolic volume changes (atrial volume in yellow line) with severe right atrial dysfunction (RA-EF: 29%). In C-D are reported end-systolic and end-diastolic 4CH view cine-SSFP images of a 51 years old male with extended ECD cardiac involvement (total localization volume 81 ml) and right atrial pseudomass of 17 ml with minimal thickening of free wall of right atrium, with preserved contractility of atrial wall and systo-diastolic modification of the volume appreciable by orange line contouring with subsequent preserved function (RA-EF: 49%)

In patients with right atrial pseudomass (14 of 29, 48%), right atrial ESV was not significantly different as compared with patients without (*p* = 0.77). However, right atrial EDV was significantly higher (EDV/BSA: 18 [IQR, 10–25] mL/m^2^ vs 10.6 [IQR, 7–12] mL/m^2^, *p* = 0.04), and atrial ejection fraction was significantly lower (RA-EF: 45 [IQR, 29.7–60] % vs 56 [IQR, 52–69] %, *p* = 0.03). Of note, in the majority of patients (10 out of 11, 91%) with a right atrial pseudomass volume > 20 mL a depressed right atrial EF (< 46%) was depicted, considering a normal range of 52.9 ± 6.7% [19] (Fig. 5).

**Fig. 6:**
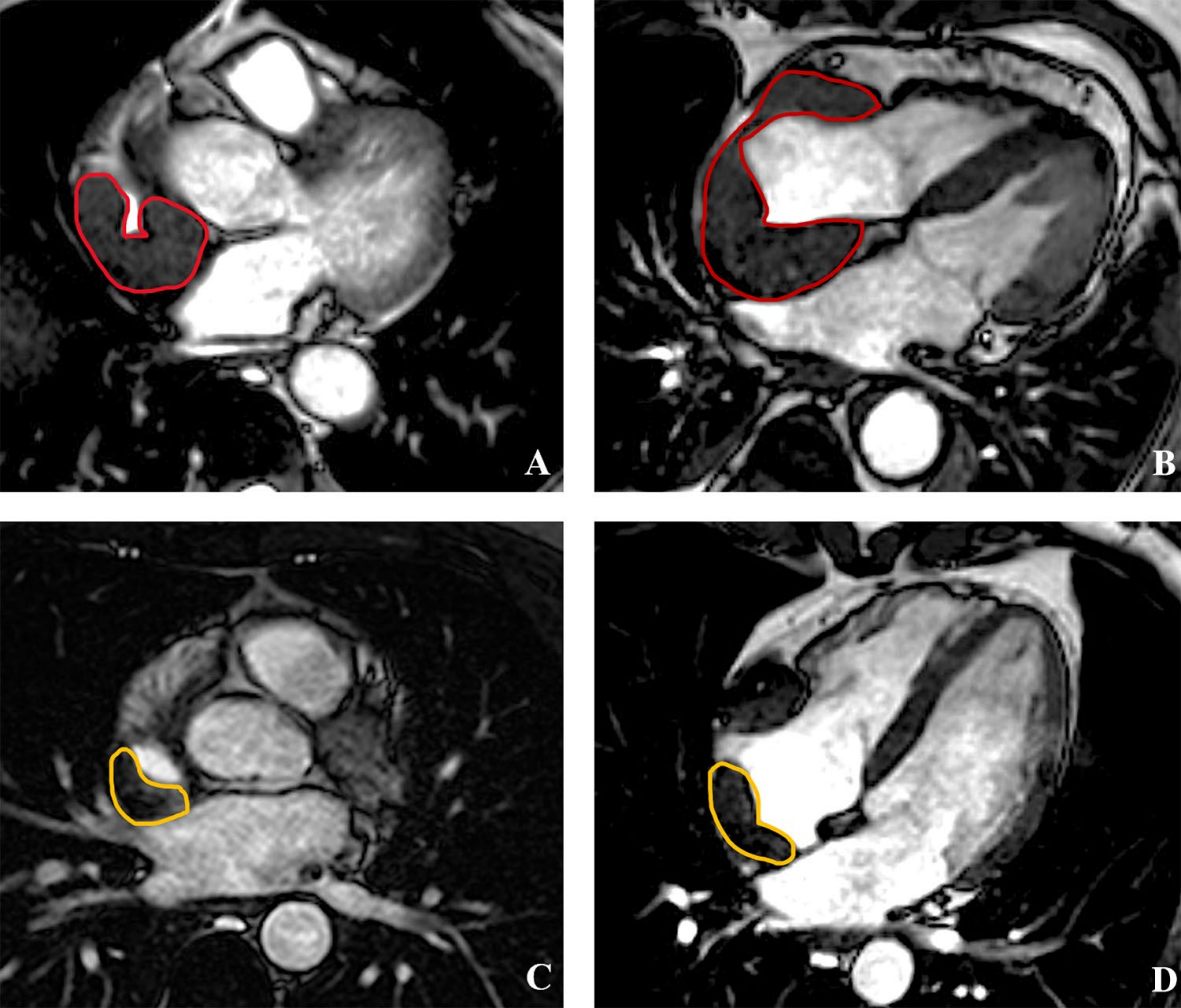
CMR images of right atrial ECD localization causing different degree of superior vena cava stenosis. In **A** and **B** are reported axial cine-SSFP images of a 48 years old female patient with right atrial pseudomass for ECD localization accounting for 38 mL (**B**) causing moderate (75%) stenosis of the superior vena cava (**A**). In **C** and **D** are reported axial cine-SSFP images of 51 years old male ECD patient showing right atrial pseudomass of 24 mL (**D**) and mild infiltration of the superior atrio-caval junction resulting in 30% stenosis of superior vena cava lumen

Vena cava stenosis was associated with bulky right atrial pseudomass. Specifically, median pseudomass volume in presence versus absence of superior vena cava stenosis was 37.8 [IQR, 24.8–38.6] mL vs 4.8 [4–5.9] mL (*p* = 0.002); whereas median pseudomass volume in presence versus absence of inferior vena cava stenosis was 37.9 [33–39] mL vs 5.95 [4.5–7] mL, (*p* = 0.04) (Fig. 6).

In the subgroup of ten patients with mapping data availability, native T1 mapping values and ECV values were slightly increased in those with ECD cardiac localization (native T1 mapping: 1067 [IQR, 1059–1098] ms vs 1027 [IQR, 1019–1042] ms, p = 0.04; ECV: 31 [IQR, 30–32] % vs 26 [IQR, 25.9–26.9] %, p = 0.017).

## Discussion

ECD is a rare multisystem histiocytosis associated with a poor prognosis, especially when the cardiovascular system is involved [20]. Cardiovascular ECD manifestations are the consequence of peri-adventitial infiltration by foamy histiocytes, with consequent development of peri-vascular and pericardial lesions [21]. Based on current available evidence, cardiovascular involvement in ECD is considered not to significantly affect cardiac function and remodeling [7]. However, systematic studies with adequate and homogeneous imaging modalities investigating the association between ECD cardiac localization and myocardial structural remodeling or cardiac chamber function are lacking.

In agreement with previous studies [2, 4, 10, 11], in our work we confirmed that right AV groove infiltration with right coronary artery encasement is the most common ECD cardiac localization. In addition, we observed that AV groove infiltration was associated with slight diffuse myocardial edema and variable degree of myocardial fibrosis with coronary distribution. LGE was characterized by non-dense fibrosis with transmural involvement in all but two patients showing inferior myocardial infarction involving 6% and 12% of the left ventricular wall. The rate of myocardial infarction found in our population (9%) is in-line with previous histologic data (11%) [21].

The most important finding of our study is that, regardless of the amount of AV pseudomasses, coronary artery encasement, due to ECD localization, seems responsible for various degrees of myocardial fibrosis. This finding substantially differs from what had been previously reported in the English literature where absence of cardiac scars or presence of only patchy LGE with non-coronary distribution was associated with cardiac ECD involvement [7, 11]. These differences could be explained by the lower rate of observed coronary encasements [7, 11], and by the different method for LGE analysis. Indeed, in the aforementioned studies [7, 11], a pure qualitative assessment affected by reader subjectivity and experience and the research of only dense scars, might be responsible for this discrepancy.

The localization and the transmural distribution of myocardial non-dense LGE we found in our population suggest ischemia as the first etiological hypothesis. Extrinsic compression or direct infiltration of coronary artery walls by histiocytes may induce vascular stenosis and increase coronary wall stiffness, thus impacting on coronary hemodynamic and promoting extracellular matrix remodeling processes in their perfusion territories. This mechanism could explain the accumulation of regional interstitial fibrosis with coronary distribution, as observed in our study population. Interestingly, a *postmortem* case report by Vaideeswar et al. [22] described an extensive infiltration of epicardial coronary arteries and evidence of critical arterial stenosis by transmural intimal plaques composed of large aggregates of foamy macrophages and lymphocytes, merged to peri-coronary ECD infiltrates. Moreover, coronary vessels infiltration by pathological histiocytes could lead to increased coronary artery stiffness and endothelial dysfunction even in the absence of morphologically critical stenoses. As a consequence, the impaired vasodilation in response to the need of increased blood supply, could also be responsible for myocardial ischemia. The potential impact of peri-coronary inflammatory infiltration on downstream perfusion is supported by recent findings from Nomura CH et al. [23] who documented reduced coronary flow reserve in patients with peri-coronary fat inflammation, even in absence of non-obstructive coronary disease.

It could be speculated that the ischemic mechanism is intrinsically associated with the inflammatory process underlying myocardial fibrosis in ECD patients. Indeed, ECD lesions were demonstrated to be characterized by spontaneous production of tumor necrosis factor-α (TNF-α), interleukin-6 (IL-6), and interleukin-8 (IL-8) [24], responsible for histiocytes’ recruitment [25]. Remarkably, IL-6 was found in vascular endothelial cells not only within ECD lesions, but also in the healthy contiguous tissues [24]. T2 signal is a well-known noninvasive MR biomarker of inflammatory infiltration and edema [26]. Indeed, specifically in ECD patients with cardiac involvement, we observed diffuse myocardial edema as disclosed by an increased T2-STIR ratio. Although the complex pathogenetic mechanisms underlying ECD are still unclear, myocardial edema might be the consequence of regional pro-inflammatory cytokine production by tissue infiltrates. Moreover, the pro-inflammatory activity in peri-adventitial coronary ECD lesions may cause a chronic inflammatory state in downstream coronary circulation, which could be responsible for pro-fibrotic pathways induction in the corresponding myocardial territories. Notably, in a previous work we demonstrated that circulating levels of chemokine ligand 18 (CCL18), which is involved in the collagen production and fibroblast proliferation, is higher in ECD patients compared to controls [27]. Additionally, we recently reported a case of myocardial infiltration by foamy cells in a patient with ECD and cardiac involvement [17].

Our findings are further corroborated by mapping results, which demonstrated diffuse alteration of native T1 values and diffuse expansion of ECV, in patients with ECD-related cardiac disease, with higher values in the posterior-septal and inferior walls in those with right peri-coronary infiltration.

Myocardial fibrosis is a known prognostic CMR biomarker, being associated with the development of ventricular arrhythmias, cardiac remodeling, and heart failure [28, 29]. However, the prognostic value in our cohort of patients was not investigated. Early identification of myocardial fibrosis in patients with ECD might be useful to improve patients’ risk stratification and to tailor treatment.

In-line with previous studies [4, 7, 11] biventricular function was preserved in all our patients. This observation might be related to the absence of diffuse myocardial infiltration, as opposed to other infiltrative disorders.

In our study, we observed right atrial pseudomasses in 14 (48%) patients. This finding is in-line with previous data, in which it was reported with a frequency between 31 and 69% [10, 12, 19, 30–32]. The increase in its amount was associated with reduced atrial contractility, with significant impairment in patients with right atrial pseudomasses. Right atrium dysfunction could potentially impact on arrhythmias onset and heart failure development [33].

Bulky right atrium pseudomasses (median mass volume: 34 mL) were associated with vena cava stenosis. In only two patients we found superior vena cava stenosis in absence of right atrium involvement. Although only few cases are reported worldwide [21, 34], in our cohort we found superior and inferior vena cava involvement in 38% and 24% of patients, respectively. This could be considered a consequence of the atrio-caval compression by the right atrial pseudomass rather than the extent of the myocardial infiltration to the surrounding vessels, as already observed at histology [34, 35].

We observed pericardial involvement and infiltration of thoracic aorta in 28% and 59% of our patients, respectively, in-line with previous reports [10, 12, 30–32]. No association between these findings and myocardial remodeling was found in our study.

Our study has some limitations. First, the sample size is relatively small. However, ECD is a very rare condition, and all our patients were studied homogeneously with the same imaging modality. Second, data about interobserver reliability lack. Nevertheless, all measurements were taken with a quantitative and semiquantitative approach to overcome subjectivity of visual interpretation, and were obtained in consensus by two readers with different experience. Finally, mapping data are available only in a subgroup of patients. Future studies aiming at assessing the reversibility of these findings after targeted and anti-inflammatory therapies [16] would be useful to improve patients’ management and risk stratification.
